# Characterization of *phoA*, a Bacterial Alkaline Phosphatase for Phi Use Efficiency in Rice Plant

**DOI:** 10.3389/fpls.2019.00037

**Published:** 2019-02-25

**Authors:** Babu Ram, Dhirendra Fartyal, Vijay Sheri, Panditi Varakumar, Bhabesh Borphukan, Donald James, Renu Yadav, Arun Bhatt, Pawan K. Agrawal, V. Mohan M. Achary, Malireddy K. Reddy

**Affiliations:** ^1^Crop Improvement Group, International Centre for Genetic Engineering and Biotechnology, New Delhi, India; ^2^Department of Biotechnology, Uttarakhand Technical University, Dehradun, India; ^3^Department of Biotechnology, Govind Ballabh Pant Institute of Engineering and Technology, Pauri Garhwal, India; ^4^National Agricultural Science Fund, Indian Council of Agricultural Research, New Delhi, India

**Keywords:** *phoA*, weed control, phosphite, phosphorus use efficiency, rice

## Abstract

Fertilizers and herbicides are two major components in the agriculture system for achieving crop productivity. Massive use of orthophosphate fertilizers and herbicides poses threats to phosphate reserves and aids the evolution of herbicide tolerant weed biotypes. Phosphite (Phi), a phosphate analog, has been proposed as more beneficial than traditionally used phosphate fertilizers and herbicides in the agriculture. We developed *phoA* overexpressing transgenic rice that minimizes the phosphate loss and contributes to weed management in the agriculture. The *phoA* rice lines showed improved root, shoot length and total biomass production under phosphite conditions. Additionally, the complete phenotype and productivity of *phoA* lines under the phosphite treatment attained was similar to that of plants under phosphate sufficient condition. The Phi metabolizing properties of the *phoA* overexpressed lines improved under the Phi application and phi treatment enabled controlling of weeds without compromising the yield of transgenic rice plants. Our results indicated that *phoA* alone or in combination with other Phi metabolizing gene(s) can possibly be used as an effective ameliorating system for improving crop plants for phi-based fertilization and weed management strategy in the agriculture.

## Introduction

Phosphorus is one of the major essential macronutrients required for the growth of plants. It attributes almost 0.2% of the plant’s dry weight and remains a part of DNA, RNA, phosphoproteins, phospholipids, sugar phosphates, enzymes and energy-rich phosphorus compounds such as ATP and NADP (Schachtman et al., 1998). It also plays an important role in numerous key biochemical reactions, cellular signaling and plant defense mechanisms. Phosphorus consumption has dramatically increased over past two decades to meet the growing agricultural demands. The phosphate-based fertilizers are completely dependent on rock phosphate, which is the non-renewable resource available on the earth. It was estimated that rock phosphate would exhaust in the next 80–400 years if the current consumption rate is maintained (Smil, 2000; Vaccari, 2009; Van Kauwenbergh, 2010). Although 90% of mined rock phosphate is used to make fertilizer, 80% of the applied fertilizer becomes unavailable for plant absorption due to its high reactivity with soil cations (Fe, Ca, Mg) and conversion of unavailable organic forms by soil microbial flora (Syers et al., 2008). The available 20% of applied P-fertilizer in the crop field is not sufficient to meet the expected global productivity and it also leads to excess use of fertilizer. This not only increases the cost of production but also leads to the eutrophication of freshwater bodies around the world (Schindler, 1977; Sharpley et al., 1994).

Inefficient use of P fertilizer in farming further intensified weed problems in the agricultural field, as they compete with the crops for nutrition, space and light and thus reduce agricultural productivity (Buhler, 2002). Weeds limit the crop resources and contribute 31.5% total crop yields loss in India (Bhan et al., 1999), 30–40% rice production in Sri Lanka (Abeysekera Anuruddhika, 2001) and 10 million tons of rice in China annually (Ze, 2001). Herbicides are commonly used to control weeds. However, continuous overuse of herbicides multiple times in a growing season increases the potential risk of evolution of resistant weeds, which has become a major concern in agriculture globally. Further, the higher dose of herbicide additionally causes concern to agricultural land and environment (Owen and Zelaya, 2005; Bhat and Chopra, 2006). About 375 resistant weed biotypes have been identified in different regions of the world against commonly used herbicides (Heap, 2012). Therefore, the inaccessibility of P fertilizer and the emergence resistance biotypes are major challenges in modern agriculture. To overcome these problems, it becomes necessary to find out an alternative route for the development of novel environment-friendly herbicides with a lower risk of evolved resistance.

Phosphite (Phi), even if similar in structure and mobility, cannot be metabolized by plants as a source of phosphate (Sukarno et al., 1993; Carswell et al., 1996; Forster et al., 1998), since it is a phytotoxic compound and inhibits overall plant growth at a higher dose (Achary et al., 2017). Many microorganisms have been identified to catalyze the oxidation of Phi into P and use it as a sole Pi source for plant growth (Ohtake et al., 1996). Studies reveal that in *P. stutzeri*, WM88 strains utilize Phi as a phosphate source, and the protein was later identified as ‘phosphite dehydrogenase’ (PtxD) and could oxidize Phi into Pi using NAD as a cofactor (Metcalf and Wolfe, 1998; Costas et al., 2001). The *ptxD* gene was further characterized in the overexpressing transgenic tobacco, *Arabidopsis* and rice plants which showed the Phi metabolizing characteristics by the transgenic plants as the sole P source for growth and development (Lopez-Arredondo and Herrera-Estrella, 2012; Manna et al., 2016). Recent studies involving *E. coli phn* mutants identified a BAP (bacterial alkaline phosphatase) enzyme that was involved in Phi oxidation (Yang and Metcalf, 2004). The BAP encoded by the *phoA* gene harboring Phi metabolizing properties has so far not been utilized in transgenic approaches. The present study is the first report to characterize the *phoA* gene from *E. coli*, transform it into rice and study the metabolizing activity using Phi as the sole Pi source for plant growth, development and metabolism.

## Materials and Methods

### Purification of Recombinant Protein and *in vitro* Enzyme Activity Assay

The rice codon-optimized *phoA* gene was chemically synthesized (Life Technologies, United States) along with *Nco1* and *NotI* sites and was cloned into pET28a. The *E. Coli* BL21 (DE3) harboring the pET28a-phoA recombinant were allowed to grow in liquid LB medium till OD_600_ and were induced with 1 mM Isopropyl β-D-1-thiogalactopyranoside (IPTG) at 37°C for 4 h. The recombinant protein was purified near to the homogeneity by Ni-NTA affinity chromatography performed at 4°C. The PhoA recombinant protein was confirmed by western blotting using anti-His antibody.

### Growth Inhibition Assay of *E. coli*

The *E. Coli* BL21 (DE3) containing either *phoA* gene or empty pET28a(+) was grown separately in 10 ml liquid M9 minimal medium. At absorbance OD_600_ 0.5, induced with 1 mM IPTG and further allowed to grow for 2 h. Following induction for 2hr, varying concentrations of Phi (100, 200, 250, 300, 350, 400, 450, and 500 mM) were added in culture media of both empty pET control and pET28a-phoA *E. Coli* and allowed further growth for 4 h. The cell growth was monitored at 600 mm by measuring the absorbance difference at every 1 h of incubation before and after 4 h of Phi treatment.

### Construction of Plant Expression Cassette of *phoA*

The plant expression cassette of *PhoA* gene (*OsAct2-P*+*phoA*+*OsAct2-T*) was constructed under the regulation of OsAct2 promoter and OsAct2 terminator from the Japonica rice variety Nipponbare and cloned initially in a Gateway compatible entry vector (*pL12R34-Amp*). The *phoA* expression cassette was transferred into plant transformation vector *pMDC99* (contains *hpt*^+^ gene as plant selection marker) using LR recombinase mediated gateway cloning process.

### Generation and Screening of Transgenic Plants

The *Agrobacterium* EHA105 harboring the recombinant *pMDC99-phoA* clone were used for plant transformation and all the plant tissue culture steps were performed in the CHU N6 media. The transformation was performed by immersing 21 day old embryogenic calli in the *Agrobacterium* culture (0.5 OD_600_) in the presence of 200 μM acetosyringone. The culture was incubated for 20–25 min on a rotator shaker at 50 rpm in the dark at 28°C. Following transformation, the infected calli were dried on Whatman filter paper and transferred to the co-cultivation medium and incubated at 28°C for 48 h. The calli were washed 6–8 times with 62.5 mg/L carbenicillin, 250 mg/L cefotaxime in sterile distilled water, dried on Whatman filter paper and transferred onto selection medium containing 50 mg/L hygromycin. After three rounds of selection with hygromycin for 30 days, the healthy secondary calli were shifted to pre-regeneration media containing 1 mg/L NAA, 2 mg/L kinetin, and 50 mg/L hygromycin for 10 days, and further transferred to regeneration media for the development of shoot for 30 days. The young regenerated shoots were transferred into half-strength MS medium for rooting and followed by hardening under greenhouse condition.

### Molecular Conformation and Identification of Transgenic Lines

Genomic DNA was isolated from putative transgenic and wild-type (*wt*) plants by CTAB method (Dellaporta et al., 1983) and initially screened for the presence of phoA with the help of PCR using sequence-specific *phoA* primers (Supplementary Table S1). The PCR conditions performed using 200 ng genomic DNA with initial denaturation at 95°C for 5 min; 30 cycles of 95°C for 1 min, 58°C for 30 s, 72°C for 1 min, and the final extension at 72°C for 10 min.

The PCR positive transgenic lines were further confirmed by Southern blotting using 10 μg RNAse treated pure genomic DNA from *wt* and transgenic lines. The Genomic DNA was separately digested with Bcl1 restriction enzyme and the restricted DNA fragments were electrophoresed on 0.8% agarose gel for 18 h at 30 V and subsequently transferred to a positively charged nylon membrane using the capillary method. The membrane was sequentially hybridized with phoA DIG-labeled gene probe and proceed for hybridization and post-hybridization and signal detection following manufacturer’s instruction (Roche Diagnostics, Germany).

### Transgenic Expression Analysis of *phoA* Gene

The expression analysis of the *phoA* gene was performed by extracting total mRNA from 30 days old control and transgenic plants using the TriZol method. The fomide-formaldehyde heat denatured 15 μg total RNA from rice line were fractionated on 1% formaldehyde-denaturing agarose gel and subsequently transferred onto a positive nylon membrane. The hybridization probe was synthesized from *phoA* coding region (660bp) was amplified by using primers specific to *phoA.* All further steps, including membrane washing and detection, were followed according to the supplier’s instructions (Roche Diagnostics, Germany).

The expression of phoA transgene was performed semi-quantitative real-time PCR (semi-quantitative RT-PCR) using gene-specific primers (Supplementary Table S1). A total of 1 μg RNA from control and transgenic lines were used for the synthesis of cDNA fragment using Verso cDNA synthesis kit. The PCR conditions performed with an initial denaturation 94°C for 1 min, 58°C for 1 min, and 72°C for 1 min for 30 cycles with initial denaturation at 94°C for 4 min using phoA gene-specific primers. The expression of the house-keeping rice eF-1α gene was used as the internal standard control.

### Seed Germination and Comparative *in vitro* Growth Analysis Under Phi Treatment

Fortnight old Japonica rice seedlings were (grown in pots) foliar sprayed with different concentrations of potassium phosphite (0, 50, 100, 150, 200, 250, 300, 350, 400, 450, and 500 mM) containing 0.1% tween 20 and allowed to grow further under the controlled greenhouse conditions. The plant growth was photographed with different day intervals (on day 4th, 7th, and 14th).

To study the ability of transgenic rice to utilize Phi as a sole phosphorus source, seeds from both *wt* and T3 homozygous transgenic lines were surfaced, sterilized and germinated on half strength MS medium with and without 10 mM Phi and grown for 15 days under controlled conditions (25°C, 12:12 hr light/dark cycle). Seeds of *wt* plants that were grown in identical conditions with and without Phi served as positive [*wt*(+)] and negative [*wt*(-)] controls, respectively. Root length, shoot length and seedling biomass was measured after 18 days of seed germination, and photographed. The root architecture and leaf greenness were observed under the stereo zoom microscope (Model: Carl Zeiss SteREO Discovery.V8) using the 1X objective lens with 45% zooming.

To determine the level of tolerance of transgenic lines to Phi concentrations, the surface sterilized *wt* seeds and transgenic lines were grown on half strength MS media containing different concentrations of Phi 0, 5, 10, 15, and 20 mM for 15 days in Petri plates under a growth chamber with a 12–12 light-dark cycle at 25°C.

### Assessment of Herbicidal Effects of Phi on Weed and Transgenic Rice

To determine the herbicide effect of Phi on the non-transgenic plant, 100 mM potassium phosphite containing 0.1% tween 20 was used for the foliar application. Transgenic lines and *wt* along with weeds (*Phyllanthus urinaria*, *Portulaca oleracea*, and *Amaranthus sp.*) were grown parallelly in the treatment tray containing soil and vermiculite mixture (2:5) for 1 month. One-month-old plants were sprayed with foliar application of Phi solution. The spraying was performed three times with an interval of 3 days between each spray and regularly photographed/recorded.

### Yield Analysis and Data Collection

The yield related experiments and phoA-transgenic plants evaluation were conducted in greenhouse conditions (14/10 h light/dark cycle illumination at 370 μEm^-2^ S^-1^ and 27 ± 1°C with 70% relative humidity) with proper biosafety levels. The yield parameters such as plant height, number of tillers per plant, panicle length, seed weight, number of seeds per panicle, 100 seed weight and total yield were recorded at the maturity stage of plant. A total 15 plants from each group were used for measurements and data collection. The pooled data were subjected to statistical analysis.

### Statistical Analysis

**FIGURE 1:**
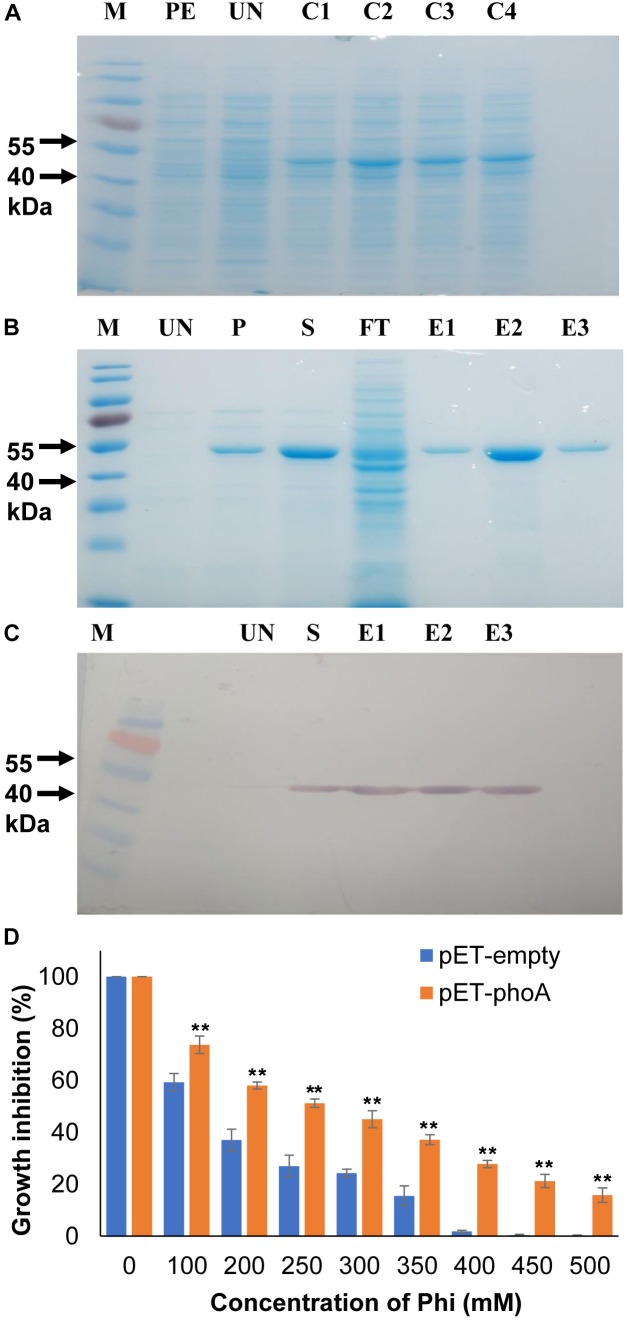
Recombinant protein expression and bacterial growth inhibition assay. **(A)** Heterologous expression of phoA **(B)** IPTG induction and purification of recombinant phoA protein by Ni-NTA method. **(C)** Western blotting confirms the expression of phoA protein in the eluted fractions. **(D)** Effect of various concentration of Phi on growth percentage of overexpression pET-phoA and empty-pET *E. coli* cells. Increase significant compared to pET empty control cells *p* ≤ 0.01 (^∗∗^). The experiment having four biological replications was repeated thrice (*n* = 12).

The quantitative experiments (Figure 1D, 4–8) were performed in duplicate or triplicate with biological replications (n). Pooled data were statistically analyzed for analysis of variance (ANOVA), followed by a least significant difference (LSD) test.

## Results

### Efficient Metabolization of Phi as Source of Phosphorus by *E*. *coli*

The phoA protein was initially functionally validated in the heterologous overexpressing *E. coli* system using M9 minimal media along with the increased phi concentrations (100–500 mM) as the sole phosphorus source. The growth of phoA over-expressing and empty pET28a (+) *E*. *coli* cells were decreased significantly (*p* ≤ 0.01) as compared to corresponding untreated cells at concentrations 100 mM and above (Figure 1D). Furthermore, the growth of phoA overexpression cells were significantly (*p* ≤ 0.01) higher compared to empty control cells along with all the Phi treatment concentrations. In addition to the above fact, the overexpressing PhoA cells also showed a significant (*p* ≤ 0.01) survival rate compared to pET control empty cells within the individual treatment group.

To monitor the kinetic properties of the phoA protein, the *phoA* gene was over-expressed with the pET28a (+) system and the recombinant phoA protein was purified near to homogeneity following Ni-NTA based affinity chromatography. The recombinant phoA was electrophorized and visualized on a 12% sodium dodecyl sulfate (SDS) polyacrylamide gel and western blotting, which corresponded to 49.5 kDa protein (Figure 1A–C).

### Generation of Transgenic Rice Lines and Molecular Analysis

**FIGURE 2:**
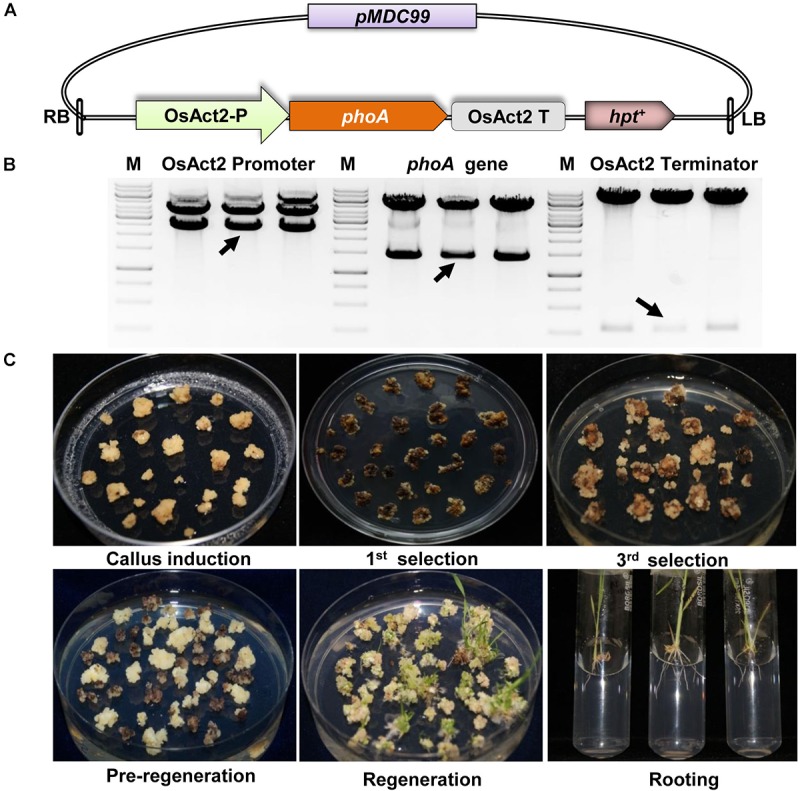
Construction of *phoA* expression cassette and plant transformation. **(A)** Schematic representation of full expression cassette of *phoA* gene. **(B)** Restriction digestion confirmation of Act2 promoter, phoA gene and Act2 terminator in pMDC99. **(C)** Various stages of plant tissue culture method for the generation of transgenics plant.

**FIGURE 3:**
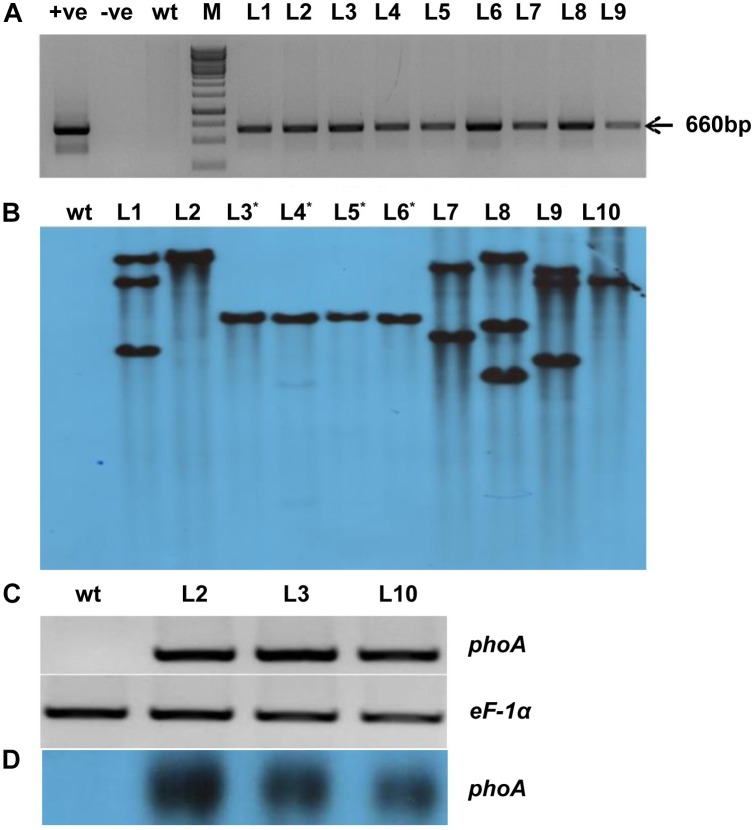
Molecular analysis of transgenic lines overexpressing *phoA.* **(A)** PCR amplification of bacterial alkaline phosphatase (*phoA*) gene using gene-specific primers; *wt* indicates wild-type; +Ve and –Ve represents positive plasmid and negative control; T0 transgenic lines L1-L09. **(B)** Southern blot analysis of ten T2 transgenic lines showing single and multiple copy insertion of the transgene. **(C,D)** Semiquantitative RT-PCR and northern blotting showing overexpression of transgenic lines (L2, L3, and L10) as compared to *wt*. The rice *eEF-1α* used as a reference control for RNA.

The codon-optimized 1416 bp *phoA* gene from *E. coli* was used by replacing rare codon for higher level expression in rice (Supplementary Figure S1). The synthetic *phoA* gene was cloned between the constitutive *OsAct2* promoter and *OsAct2* terminator in EV-1 and the plant expression cassette was cloned into *pMDC99* plant transformation vector (Figure 2A,B). The *Agrobacterium* EHA105 strain carrying the recombinant *pMDC99-phoA* expression cassette was transferred into Japonica rice cultivar var Nipponbare (Figure 2C). Following the rice tissue culture protocol, a total of 32 putative rice transgenic lines were generated. The putative transgenic lines were initially screened for the integration of *phoA* gene and the PCR positive lines (Figure 3A) were germinated on hygromycin and allowed multiplication up to T3 generation. The T3 homozygous transgenic line along with *wt* plant was subjected to Southern analysis to identify the independent transgenic event along with the transgene copy number.

All the (L1-L10) transgenic lines showed one or more hybridization bands, however, there was no southern positive band in the *wt* control plant (Figure 3B). The three-independent and single transgene integrated lines (L2, L3, and L10) were selected for their expression study by semi-quantitative RT-PCR and northern analysis (Figure 3C,D). All the selected lines showed expression level of *phoA* gene while there was no expression found in the control *wt* plant (Figure 3C,D). Furthermore, among the transgenic lines, the L2 showed a relatively high level of transgene expression followed by L3 and L10 lines.

### Effect of Varying Concentration of Phi on *phoA* Transgenic Rice

**FIGURE 4:**
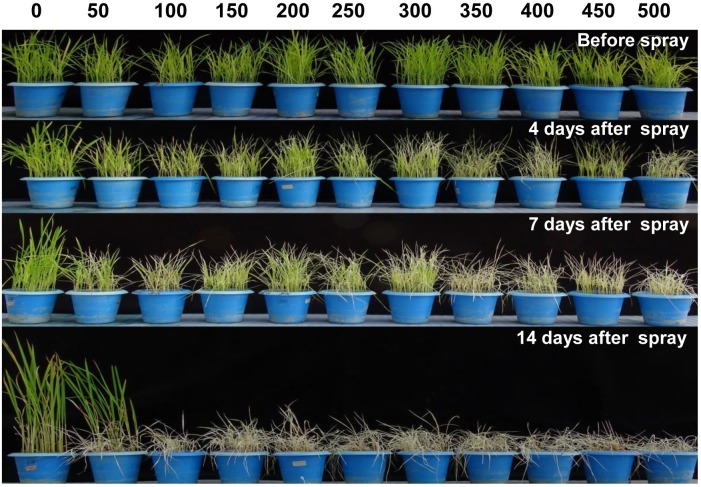
Evaluation of phytotoxicity of Phi on *wt* rice seedling. Fortnight old *wt* rice seedlings were foliar sprayed with a range of Phi concentration 50–500 mM. The symptom was photographed on 4nd, 7th, and 14th days after spray. The experiment having three biological replications was repeated twice (*n* = 6).

In order to standardize the toxic concentration of Phi on *wt* rice seedlings, 14 days old rice seedlings were foliar sprayed with 50–500 mM potassium phosphite (Figure 4). The *wt* control plants without Phi spray appear normal, whereas plants sprayed with Phi showed symptoms of leaf chlorosis and the symptom appeared at 50 mM and further Phi concentrations. The intensity of chlorosis was dose-dependent and was increased with higher concentrations of Phi. The present study revealed that 50 mM Phi is the optimum and beyond this concentration, seedlings completely die within 14 days of spraying (Figure 4).

**FIGURE 5:**
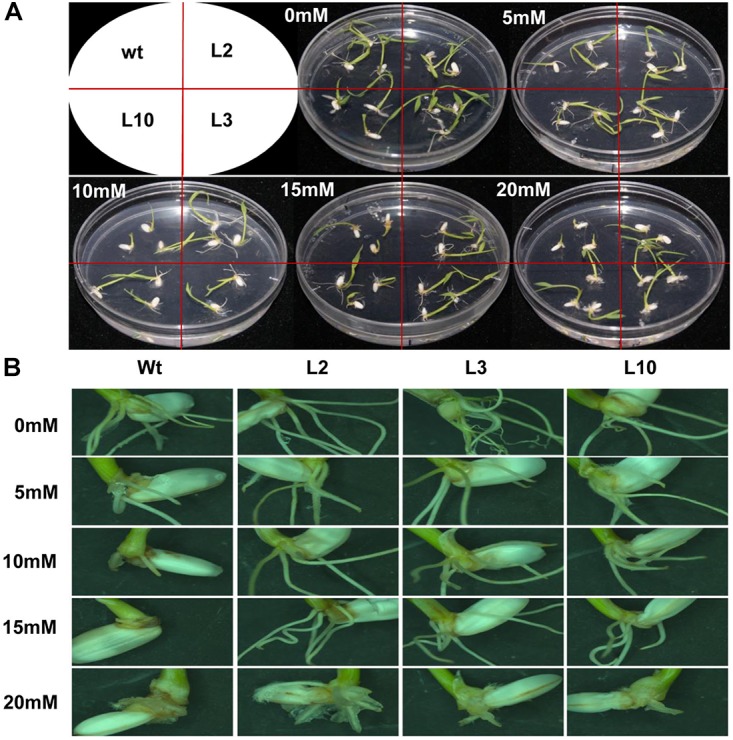
Effects of Phi treatment on root morphology of *wt* and *phoA* transgenic lines. **(A)** The growth of *wt* and transgenic lines on varying concentration of Phi (5–20 mM). The *wt* and transgenic lines were continuously grown for 12 days on half MS medium containing Phi, deficient in Pi. **(B)** Comparison of root hair or root formation in *wt* and transgenic lines. The experiment having three biological replications was repeated twice (*n* = 6).

The Phi metabolizing properties of phoA transgenic rice were evaluated by germinating rice seed from *wt* and phoA transgenic lines (L2, L3, and L10) on phosphate-free half MS medium supplemented with varying concentrations of potassium phosphite (0, 5, 10, 15, and 20 mM). Here we observed that with the increased Phi concentration, *wt* plants showed a negative effect on the root growth and architecture that followed a dose-dependent phenotype (*P* ≤ 0.01) at 10 mM and higher concentrations. However, the root growth of transgenic lines showed normal root morphology up to 15 mM Phi concentration and was marginally inhibited at 20 mM. (Figure 5A,B). Similarly, the shoot development was significantly reduced (*P* ≤ 0.01) in *wt* plants at 15 mM Phi, whereas transgenic plants showed normal phenotype at 15 mM and no effect up to 20 mM Phi concentration. The results indicate the effective metabolizing properties of Phi by the phoA-transgenic lines used as a sole phosphorus source in this experimental condition and showed comparatively improved root and shoot length and total biomass production at each of the given concentrations compared to *wt* control plant (Supplementary Figure S2).

**FIGURE 6:**
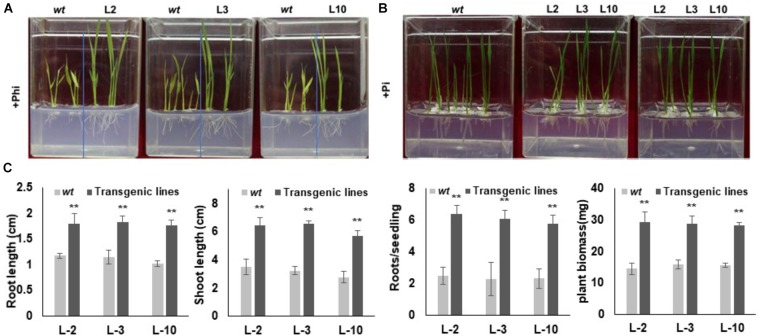
Seedling morphology of transgenic rice lines under Phi and Pi condition. **(A)** Comparative growth morphology of *wt* and transgenic lines under Phi condition. The growth of *wt* and T3 *phoA* lines in half MS medium supplemented with 10mM Phi, deficient in Pi. **(B)** Transgenic and *wt* seeds were grown in half MS medium containing Pi, without phi. **(C)** Comparative analysis of root-shoot length (cm) and fresh weight (mg) of *wt* and transgenic lines grown under Phi condition. Increase significant compared to control *p* ≤ 0.05 (^∗^) or 0.01 (^∗∗^). Data represent mean ± standard deviation (*n* = 9). The experiment having three biological replications was repeated thrice.

In order to assess the response and identification of the best transgenic events, selected phoA transgenic lines were separately germinated on 10 mM Phi containing phosphate free half the strength of MS medium along with *wt* control seeds for 20 days (Figure 6A). A similar set of experiments were conducted using 10 mM Pi containing half the strength of MS medium as a reference or positive control. No significant morphological difference between the L2, L3 and L10 transgenic events were observed in the Pi germination media, which were comparable to that of *wt* control (Figure 6B). In contrast to the Pi treatment, *wt* plants showed leaf chlorosis, stunted root-shoot growth and reduced biomass in Phi growth medium (Figure 6A). On the other hand, the transgenic lines with overexpressed *phoA* effectively metabolize Phi as P source without affecting the plant growth and development, and showed significantly (*P* ≤ 0.01) higher root-shoot length and higher biomass compared to *wt* control plants (Figure 6A–C).

### Herbicidal Effect of Phi on Weeds-Transgenic Crop Competition and Yield Penalty in the Transgenic Lines

**FIGURE 7:**
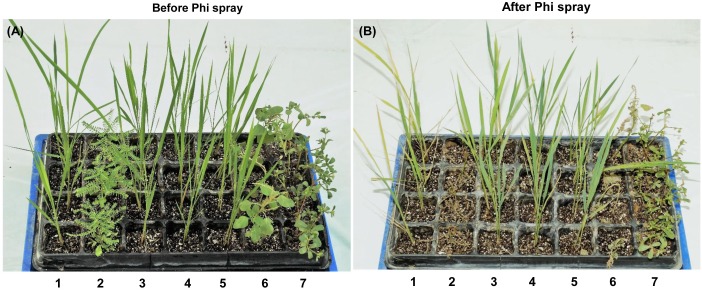
Effect of Phi on *wt* plant, phoA transgenic line and weed population. Post-emergent herbicide effect of Phi. The Figure represents the before **(A)** and after **(B)** spray of Phi on *wt* (1), weeds [*Phyllanthus urinaria* (2), *Amaranthus sp* (6) and *Portulaca oleracea* (7) and transgenic lines L2 (3), L3 (4) and L10 (5). The experiment having four biological replications was repeated twice (*n* = 8).

Weeds compete parallelly with crop plants in the crop field and increase production and processing costs. To determine the effect of Phi as a post emergence herbicide, a 1-month-old *wt* plant, phoA transgenic lines L2, L3 and L10 along with weeds (*Phyllanthus urinaria*, *Portulaca oleracea* and *Amaranthus sp.*) were foliar sprayed with 100 mM potassium phosphite (Figure 7A). The initial symptoms of leaf chlorosis were seen after the fourth day of foliar spray and it started from the leaf tips in *wt* control rice. Clear visible symptoms of leaf chlorosis were observed in the *wt* plants which were absent in the transgenic lines. Further, among the weeds *Phyllanthus urinaria* died quickly on day 3 of Phi spray, followed by *Amaranthus.* However, application of Phi at this concentration has a moderate effect on *Portulaca oleracea*, which survived even after the third round of foliar spray. This could be due to the difference in physiological and/or morphological tolerance among the weed biotype. Phi acts as a toxic compound and its effects are systematic in nature, which is well documented and demonstrated in many crop plants. Similar instances of systematic chlorosis that spread over the whole leaf were observed during the present study on weeds and *wt* control plants (Figure 7B). This also gives a confidence that Phi can be utilized as a post-emergent herbicide to kill or suppress weed growth.

**FIGURE 8:**
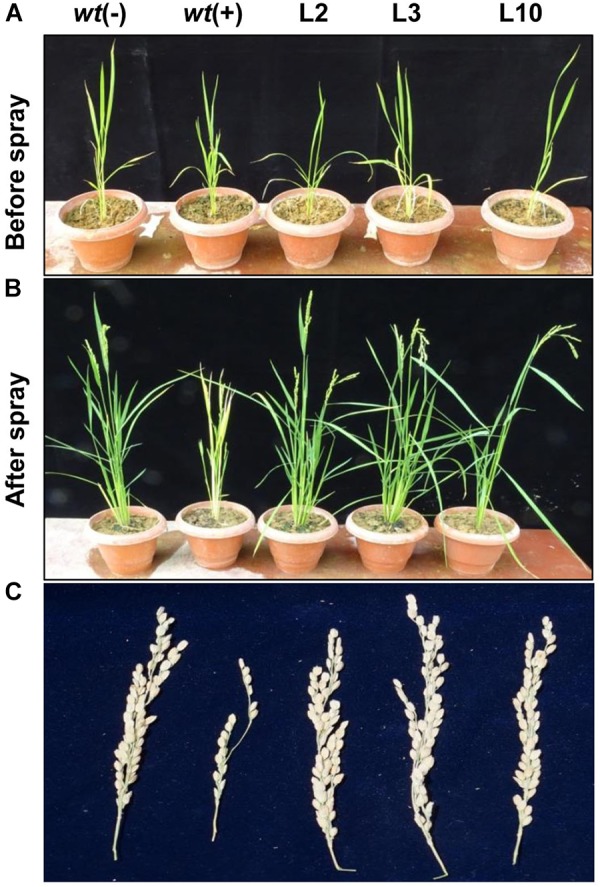
Effects of Phi on yield. **(A,B)** The phenotype difference of *wt* and transgenic lines before and after foliar application Phi 100 mM. **(C)** Yield parameters and penicle phenotype of *wt* and *phoA* transgenic lines under Phi application. The experiment having five biological replications was repeated thrice (*n* = 15).

To assess the yield penalty of *wt* and phoA-transgenic lines, the 1-month-old rice seedlings were sprayed with 100 mM potassium phosphite thrice with an interval of 3 days and the plants were allowed to grow until maturity under controlled greenhouse conditions (Figure 8A,B). No visible morphological differences were observed between the transgenic lines on Phi application and showed normal plant growth and stay-green in nature comparable to *wt*(-) control plant. However, the *wt* plants were able to survive, but showed overall stunted growth and leaf chlorosis. The transgenic lines showed normal morphology including plant height, number of panicles per plant, panicle length, and 100-grain weight (Table 1). The above yield contributing traits were highly influenced and got reduced in the *wt*(+) plant on Phi treatment. No significant variations were observed among the transgenic lines for the yield contributing traits. The study clearly demonstrated that Phi acts as a post emerging herbicide by significantly suppressing the non-transgenic plants.

**Table 1:** Yield of *wt* and *phoA* transgenic lines under control and Phi treated conditions.

	Plant height (cm)	Number of panicle/plant	Panicle length (cm)	Number of seed/plant	Seed weight (mg)	Weight per 100 grains (g)	Yield/plant (g)
*wt*(-)	59.78 ± 1.761	5.4 ± 10.548	14.22 ± 0.356	543.25 ± 34.8	21.2 ± 1.17	2.12 ± 0.084	11.56 ± 0.345
*wt*(+)	39.14 ± 0.750	2.6 ± 0.548	7.92 ± 0.303	145.26 ± 20.4	17.63 ± 0.96	1.78 ± 0.084	2.58 ± 0.383
L2	59.84 ± 2.682 (b)	5.6 ± 0.548 (b)	14.06 ± 0.288 (b)	552.62 ± 26.0	21.1 ± 1.5	2.14 ± 0.114 (b)	11.82 ± 0.370 (b)
L3	60.34 ± 0.98 (b)	5.6 ± 0.548 (b)	13.98 ± 0.277 (b)	561.63 ± 34.7	21.5 ± 1.3	2.14 ± 0.167 (b)	12.04 ± 0.581 (b)
L10	60.5 ± 1.841 (b)	5.2 ± 0.447 (b)	13.98 ± 0.370 (b)	569.75 ± 22.3	21.8 ± 1.7	2.18 ± 0.130 (b)	12.64 ± 0.230 (b)


*The experiment having five biological replications was repeated thrice (n = 15). Increase significant compared to wt control (+) p ≤ 0.05 (a) or 0.01 (b).*

## Discussion

Phosphorus is one of the major macronutrients, the scarcity of which directly affects crop health and agricultural productivity. It is a non-renewable resource, expected to exhaust in the near future due to the overuse of phosphate-based fertilizers. The decline in reserves of rock phosphorus is a major global concern due to its high consumption rate in agriculture. Looking to the issue of resistance due to the application of herbicides and cost to the environment, an alternate strategy is required to overcome the problem, without compromising crop productivity. More than two-thirds of the phosphorus applied is not utilized by the plant. They are either locked-up with soil particles or move into water bodies, affecting the soil, health and environment. The efforts to improve phosphorus use efficiency had little success for crop productivity. Phosphite, an alternate structural form of inorganic phosphate cannot be metabolized by plants since plants do not have a mechanism for its metabolism. Recently, Lopez-Arredondo and colleagues (Lopez-Arredondo and Herrera-Estrella, 2012) and Manna et al. (2016) have shown the use of phosphite as a sole phosphorus source by the overexpression of the Phosphite dehydrogenase gene from *Pseudomonas stutzeri* in transgenic plants (Lopez-Arredondo and Herrera-Estrella, 2012; Manna et al., 2016). Similarly, the bacterium *E. coli* possesses two different mechanisms to oxidize phosphite into inorganic phosphate. The enzyme C-P lyase is one of the mechanisms consisting of 14-gene *phn* operon and the second mechanism involves *phoA* locus, which encodes bacterial alkaline phosphatase (BAP). In *E. coli*, under phosphate-limiting conditions, it acts as an inorganic phosphate scavenger, releasing inorganic phosphate from the phosphate-esters of the external medium. However, studies revealed that purified BAP catalyzes the conversion of phosphite to phosphate and molecular H_2_. BAP is a phosphite dependent hydrogenase, located in periplasmic space in *E. coli*, that involves a direct transfer of a hydride from the substrate to water derived protons converts Phi to Pi (Kechao and William, 2004).

The present study involves overexpression of phoA gene of *E. coli* in Japonica rice cultivar for functionally validating the Phi use efficiency of the transgenic rice plants. For being a non-metabolizable form of P and plant’s quick uptake and distribution throughout the system, Phi interferes with the Pi metabolism pathway and suppresses the expression of starvation-induced genes including acid phosphatases (Carswell et al., 1997; Ticconi et al., 2001). Such behavior of Phi can be harnessed for utilising it as a herbicide. Moreover, phoA mediated detoxification of Phi will render it’s use as an alternative form of P fertilizer for the cultivation of transgenic rice. The accumulated Phi in the plant interferes with those biochemical pathways which use Pi as one of their substrates thus misleading the Pi-sensing mechaniary of the plants. Attenuation of Pi-starvation response further creates phosphate starvation situation inside the plants leading to phytotoxicity and suppression of plant growth (Carswell et al., 1997). Similar findings were observed in the 15 days-old seedlings sprayed with 50 mM Phi that showed optimum growth. However, the concentrations beyond this affected the survival of the plants in a dose-dependent manner (Figure 4). Similarly, Phi was found to negatively affect the growth of spinach plants, the shoot dry weight was found to decrease along with an increased Phi concentration (Thao et al., 2008a). Experiments with *Brassica rapa* var Peruviridis also yielded similar results (Thao et al., 2008b). Hirosse et al. (2012) found that the increase in the dose of Phi resulted in decreased growth, length and dry weight of sweet potato (Hirosse et al., 2012).

The codon-optimized *phoA* gene was overexpressed under the control of *Act2* constitutive promoter. We have generated 25 independent *phoA*-overexpression transgenic events, of which L2, L3, and L10 showed single transgene integration (Figure 3B). The success of crop biotechnology lies with the tissue-specific and high-level transgene expression. Rice *Act2* promoter has been proven as an efficient promoter in many monocot plants (Chengkun et al., 2009). The *phoA* transgenic rice showed normal root morphology and shoot phenotype at 10 mM Phi treatment. The result indicates the effective metabolizing properties of Phi by the *phoA*-transgenic lines used as a sole source of phosphorus and showed comparatively improved and higher root, shoot length and total biomass compared to *wt* control plant. The root, shoot and biomass of transgenic lines were significantly higher than *wt* plant. On the other hand, *wt* plant showed leaf chlorosis, stunted root-shoot growth (Figure 6). The similar morphological changes were witnessed due to the negative effect of Phi on the growth of onion, tomato, *B. nigra* and *Arabidopsis* (Sukarno et al., 1993; Carswell et al., 1996, 1997; Forster et al., 1998). Manna et al. (2016) showed that 25 mM sodium Phi concentration severely affects the root to shoot ratio of *wt* rice seedling. Our results demonstrated that *phoA* transgenic plants had overall improved physiology and phenotype compared to the *wt* rice plants when supplemented with Phi as the sole source of P. This indicated that *phoA* transgenic plants were able to metabolize Phi to Pi and use it as a P source. Therefore, phoA can be a good candidate gene for the development of a transgenic plant that utilizes Phi as a sole Pi source for its growth and development.

In order to meet the growing demand for food, a large quantity of P fertilizers have been used in agriculture globally. More than 80% of the unavailable Pi fertilizer get washed away ultimately into ocean water bodies which cannot be recovered or recycled. This not only damages the water ecosystem through eutrophication but also raises alarming P scarcity concerns in near future. Moreover, excess applied Pi fertilizer aggravates weed problems in agriculture competing for P nutrient with crop plant. Chemical based herbicide has been extensively applied in the past few decades to control or kill various weed populations in agriculture. Continuous and excess application of the same herbicide builds selection pressure on weed that leads to the evolution of a large number of herbicide-resistant superweeds. Furthermore, chemical herbicide damages both the environment and human health (Van Bruggen et al., 2018). The other mechanism by which weeds escape from the herbicidal activity is by reduced herbicide uptake or sequestering herbicide into subcellular compartments. Phi application on plants fails to stimulate phosphate starvation responses (PSRs) that leads to break the sense of Pi deficiency mechanism and produce phytotoxicity symptoms. Phi being phytotoxic in nature, can be greatly explored as weedicide since the normal plant cannot metabolize it. With an objective to roll-out use of Phi as weed control strategies, we demonstrated that Phi suppresses the growth of weed population effectively (Figure 8). A similar observation has been reported in earlier studies in the *ptxD* overexpressing transgenic lines (Lopez-Arredondo and Herrera-Estrella, 2012; Manna et al., 2016). The present finding underscored the role of the *phoA* gene and the use of Phi as a weed control strategy to suppress various harmful weeds in agriculture and add benefits to *phoA* transgenic lines. Furthermore, the strategy checks huge phosphorus loss, keeping the environment clean and reducing the burden of additional herbicide application in the field. Phi has dual advantage over conventional herbicide as Phi absorbs and transports into plants that cannot avoid its uptake and, secondly, to develop resistance against Phi, an extra gene in required in the weed genome to metabolize Phi, which may take several generations to accumulate.

Furthermore, the Phi application showed advantages in *phoA*-transgenic lines with no morphological difference recorded and the transgenic plants showed normal plant growth and physiology. The transgenic lines showed normal growth including plant height, number of panicles per plant, panicle length and 100-grain weight without compromising the yield potential of the plant. An earlier study in the overexpression *ptxD* transgenic lines showed that about 30–50% less Phi is required to attain similar productivity to that obtained by the same plants using Pi based fertilizer (Lopez-Arredondo and Herrera-Estrella, 2012). A similar observation has also been reported by Manna et al. in the overexpression *ptxD* rice transgenic lines (Lopez-Arredondo and Herrera-Estrella, 2012). Thus, there is a technical possibility to economize P use in actual field conditions.

## Conclusion

The above results clearly suggest that transgenic overexpressed *phoA* effectively utilizes Phi as a P source without compromising plant growth and development. The phoA transgenic showed a double advantage to check the overuse of ortho-phosphate based fertilizer and keep water bodies clean. Moreover, Phi based fertilization controls a variety of the weed population that leads to an advantage to the crops as well as the farmers to stop the additional herbicide use. Phi, being an excellent fungicide, has been used in many parts of the world to suppress and kill fungus pathogens which damage crop plants and this provides an additional advantage in Phi technology. To conclude, Phi based agriculture technology can not only be used as an alternative to ortho phosphorus based fertilization, but at the same time, can also act as a weedicide and a fungicide to control fungal pathogens in the agriculture field.

## Author Contributions

BR and VA developed the plant expression cassette. BR, DF, VS, BB, RY, AB, and PV involved in plant tissue culture and helped in molecular analysis of transgenic plant. BR, VA, and DJ carried out all physiological experiments. MR and PA conceived the presented idea. VA and BR designed all the experiments, critically analyzed the data, and drafted the manuscript.

## Conflict of Interest Statement

The authors declare that the research was conducted in the absence of any commercial or financial relationships that could be construed as a potential conflict of interest.

## Supplementary Material

FIGURE S1 The coding sequence of codon optimized *phoA* gene from *E. coli*.

FIGURE S2 Seedling morphology of wt and *phoA* transgenic lines under different Phi applications.

TABLE S1 List of primers used for making *phoA* plant expression cassette, cloning into the protein expression vector system, validation of transgenic plant by PCR and expression analysis.
