# Supplementary figures and images for: Characterization of phoA, a Bacterial Alkaline Phosphatase for Phi Use Efficiency in Rice Plant
Source: Front Plant Sci. 2019 Feb 25;10:37. doi: 10.3389/fpls.2019.00037 (PMC6397861; doi:10.3389/fpls.2019.00037)

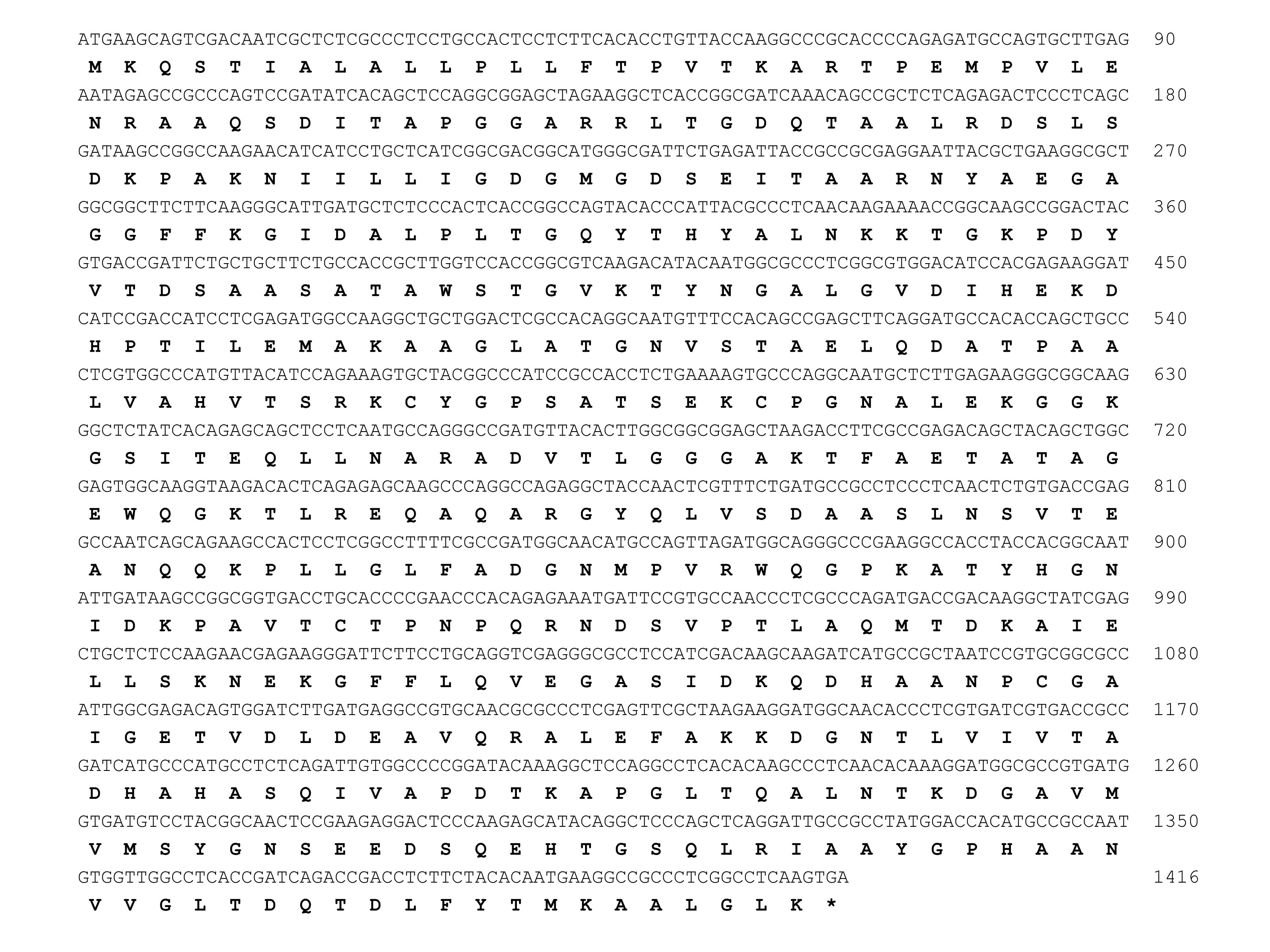

Supplement: FIGURE S1 — The coding sequence of codon optimized phoA gene from E. coli. [file Image_1.JPEG]

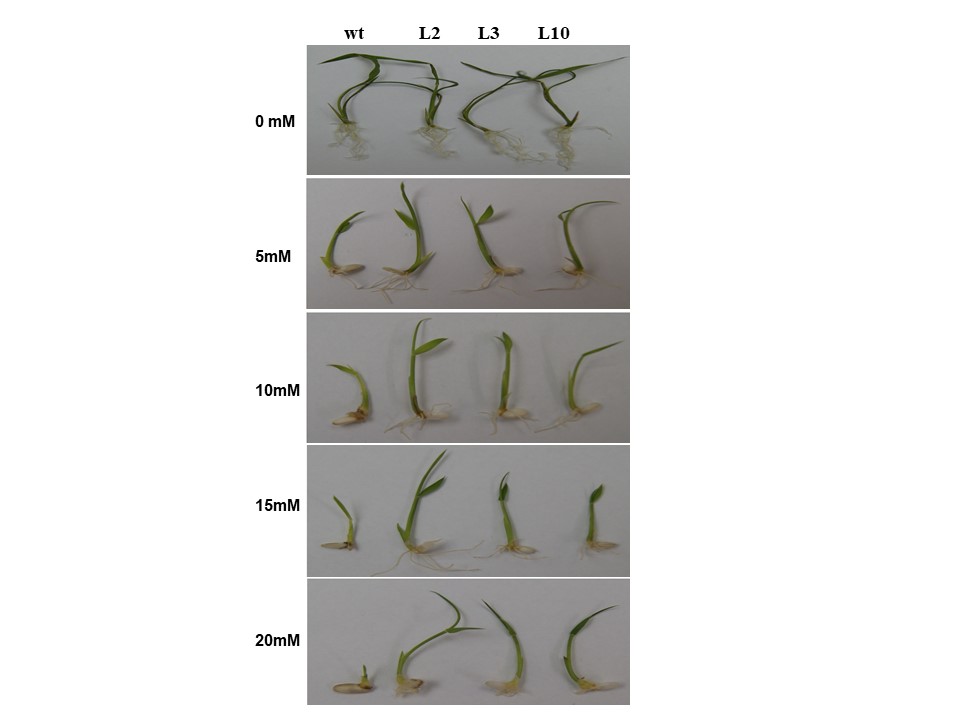

Supplement: FIGURE S2 — Seedling morphology of wt and phoA transgenic lines under different Phi applications. [file Image_2.JPEG]
