# Supplementary material for: Characterization of phoA, a Bacterial Alkaline Phosphatase for Phi Use Efficiency in Rice Plant
Source: Front Plant Sci. 2019 Feb 25;10:37. doi: 10.3389/fpls.2019.00037 (PMC6397861; doi:10.3389/fpls.2019.00037)
Supplement: TABLE S1 — List of primers used for making phoA plant expression cassette, cloning into the protein expression vector system, validation of transgenic plant by PCR and expression analysis. [file Table_1.DOCX]

**Table 1:** List of primers used for making *phoA* plant expression cassette, cloning into the protein expression vector system, validation of transgenic plant by PCR and expression analysis.

| Primer name | Primer sequence (5’------------3’) |
| --- | --- |
| OsAct2(Kpn1)F | AAGGTACCTCCATGCCTACATCAACT |
| OsAct2(Nde1)R | ACTCATATGTTATTTTAACTGATCTGCATAACAC |
| OsAct2 Ter (NotI)F | ATAGCGGCCGCATGTGGCCTAGCTGTATCT |
| OsAct2 Ter (SacI)R | GCCGAGCTCAGCTCTCAAGAATCTCAAAT |
| phoA screen F | CAAGGTAAGACACTCAGAGAGCAA |
| phoA screen R | ATTGTGTAGAAGAGGTCGGTCTGAT |
| phoA-pET (-SP)BamH1F | ATAGGATCCATGCGCACCCCAGAGATGCCA |
| phoA-pET Not1R | ATAGCGGCCGCTCACTTGAGGCCGAGGGC |
